# Immunotherapy-Induced Auto-Splenectomy in a Patient of Malignant Melanoma

**DOI:** 10.7759/cureus.25067

**Published:** 2022-05-17

**Authors:** Syed Ehsanullah, Azharuddin Muhammad, Syed Hasan, John M Richart

**Affiliations:** 1 Medicine, Washington University School of Medicine, St. Louis, USA; 2 Hematology and Medical Oncology, Saint Louis University School of Medicine, St. Louis, USA; 3 Oncology, Saint Louis University School of Medicine, St. Louis, USA; 4 Hematology and Oncology, Saint Louis University School of Medicine, St. Louis, USA

**Keywords:** newest treatment for multiple myeloma, pdl-1 inhibitor, immunotherapy-related adverse events, autosplenectomy, cancer immunotherapy

## Abstract

Immune checkpoint inhibitors like programmed cell death 1 (PD-1) and cytotoxic T-lymphocyte-associated protein 4 (CTLA-4) are biological agents that help in boosting the immune system of the body to fight against cancer cells. These checkpoint inhibitors are now being approved by the Food and Drug Administration (FDA) to treat various malignancies due to remarkable response. Here, we present a rare immune-related adverse event in a 77-year-old female with metastatic melanoma treated with ipilimumab and nivolumab, later presented with auto-splenectomy.

## Introduction

Cancer is the second leading cause of mortality in the United States [1]. Among all cancers, skin malignancy is the most common. Melanoma is the most aggressive and dangerous form of skin malignancy [1]. Cytotoxic T-lymphocyte-associated protein 4 (CTLA-4) inhibitors were first approved by Food and Drug Administration (FDA) in 2011 for the treatment of melanoma [2]. Meanwhile, programmed cell death 1 (PD-1) inhibitors were approved in 2015 by FDA for melanoma [3]. Ipilimumab is an anti-CTLA-4 agent that promotes the function and growth of T-cells by strengthening the immune system. Nivolumab is a PD-1 inhibitor that promotes the tumor-killing effects of T-cells. Ipilimumab and nivolumab have emerged as successful treatments and have demonstrated an overall survival benefit in metastatic melanoma [4]. Since immunotherapies are becoming more common, immune-related adverse events are now coming into the limelight. The adverse effects are thought to be related to the development of auto-immunity [5]. Auto-splenectomy is an extremely rare complication and adverse event of ipilimumab and nivolumab. So far, only one case has been reported in the published literature [6].

## Case presentation

A 55-year-old female with no significant past medical history was presented to the oncologist's office as she noticed that she had a lesion on the right anterior scalp during a haircut. She initially observed it but later sought medical attention when the lesion got more prominent with some bleeding and crusting. She had a shave biopsy for her scalp lesion. Pathology revealed malignant melanoma with a Breslow thickness of 2.7 mm and Clark level IV, with bleeding ulceration. She was referred for surgery. The patient underwent a wide local excision in 2012. Sentinel lymph node biopsy was negative for regional metastasis. A whole-body imaging positron emission tomography (PET) scan was also negative for metastatic disease. According to the American Joint Cancer Committee (AJCC-8) staging, her disease was graded as stage II melanoma. One year follow-up computerized tomography (CT) scan neck was unremarkable, but CT-chest revealed a 5 mm lung nodule in the left lower lobe, which was concerning for metastatic disease. Since the lesion was too small for biopsy, it was decided to monitor the lung nodule.

**Figure 1:**
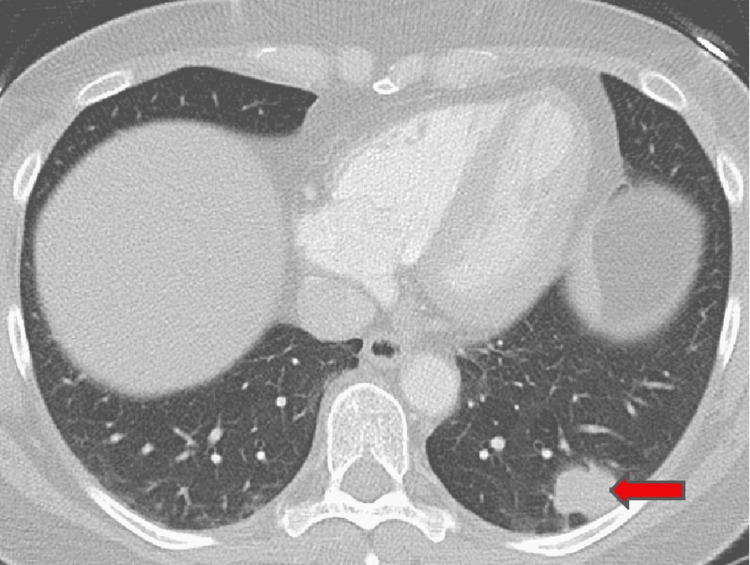
CT chest axial lung window showing irregular soft tissue density left lower lobe mass (arrow). CT: computed tomography

**Figure 2:**
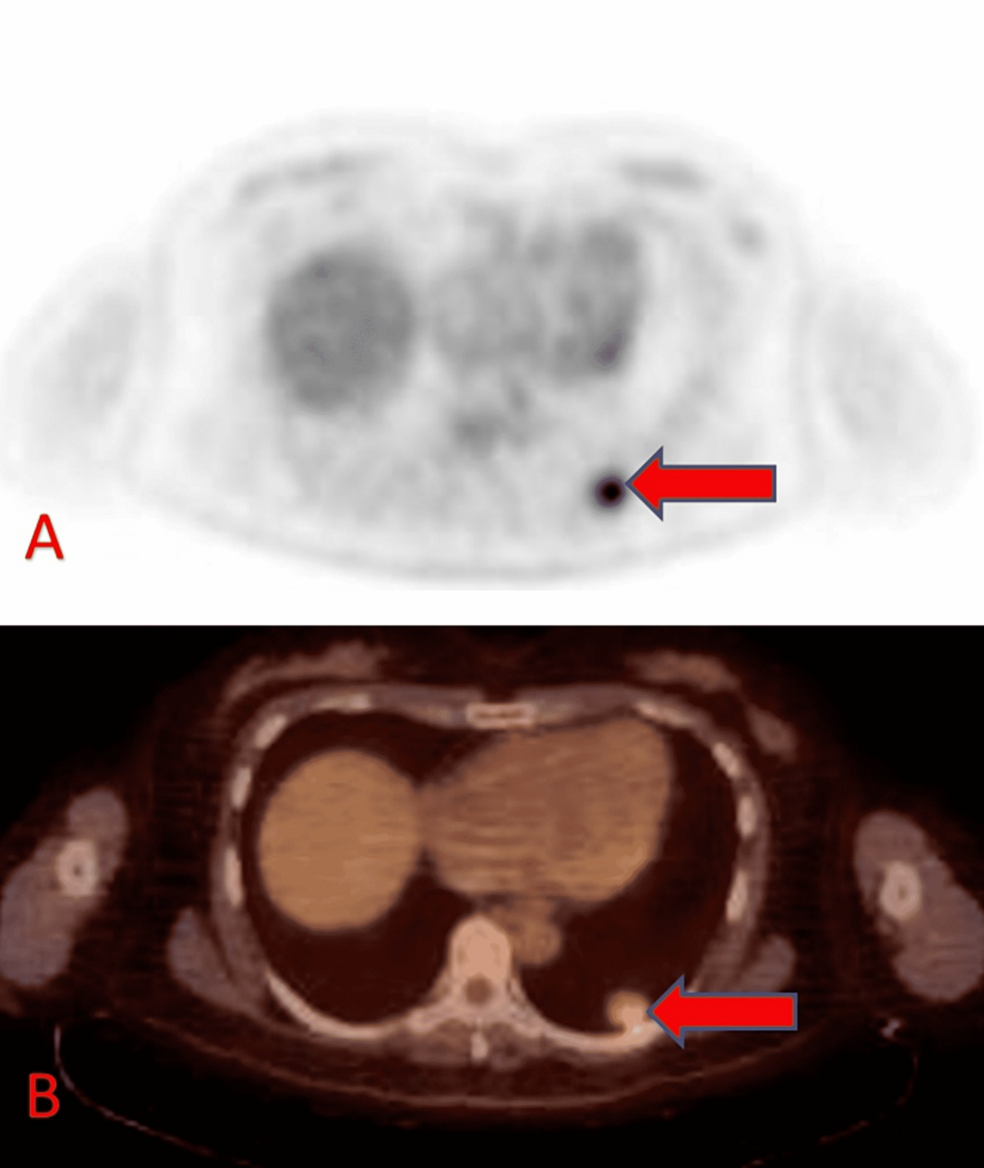
Axial FDG PET (A) and fused PET CT (B) showing moderate FDG uptake in the left lower lobe lung nodule (arrows). FDG: fluorodeoxyglucose; PET: positron emission tomography; CT: computed tomography

**Figure 3:**
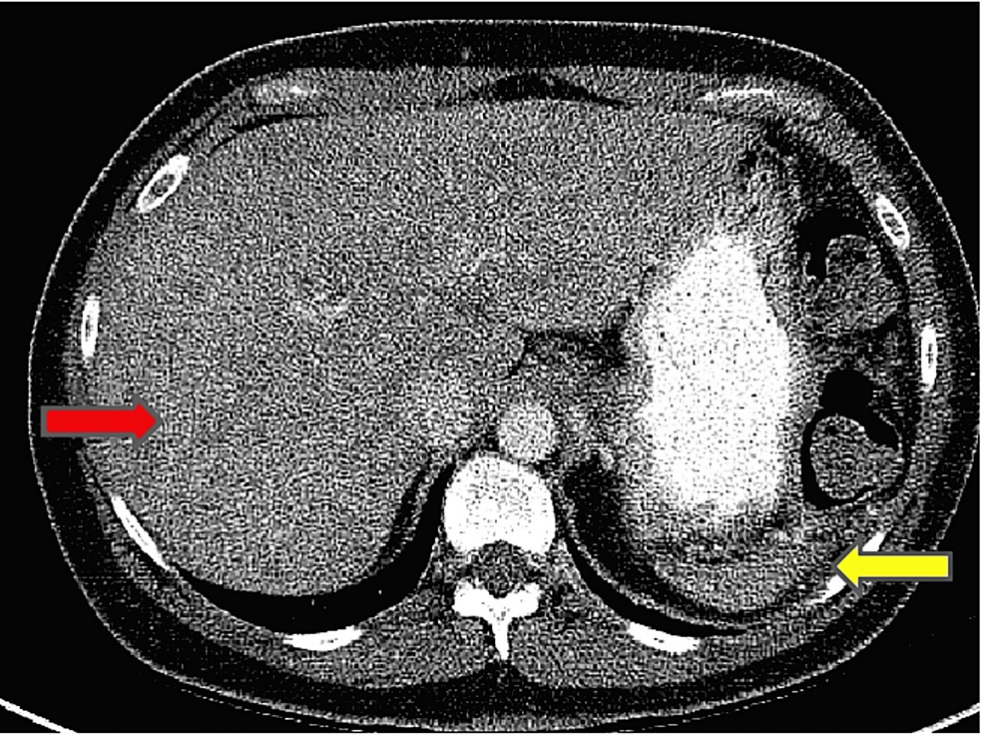
Post-therapy CT contrast axial slice showing the heterogeneous appearance of the liver secondary to innumerable hypodense hepatic lesions (red arrow). A small-sized spleen is also noted (yellow arrow). CT: computed tomography

A follow-up CT scan in 2016 revealed an increase in the left lower lobe lesion to 2.3 cm, with an adjacent 1.1 cm lesion and a 1 cm right upper lobe lesion (Figure 1). The patient underwent a CT-guided biopsy of the left lower lesion, consistent with metastatic melanoma. BRAF V600E gene mutation was negative. A whole-body PET scan demonstrated FDG avid lesion in the left lung (Figure 2). Brain MRI was negative for any brain involvement. The patient was started on ipilimumab and nivolumab, combination immunotherapy. After completing four cycles of combination therapy, the patient remained on maintenance nivolumab 240 mg every two weeks. Post-treatment CT scan of chest, abdomen, and pelvis showed decreased size of multiple pulmonary nodules, with near resolution of the right upper lobe lung lesion (Figure 3).

**Figure 4:**
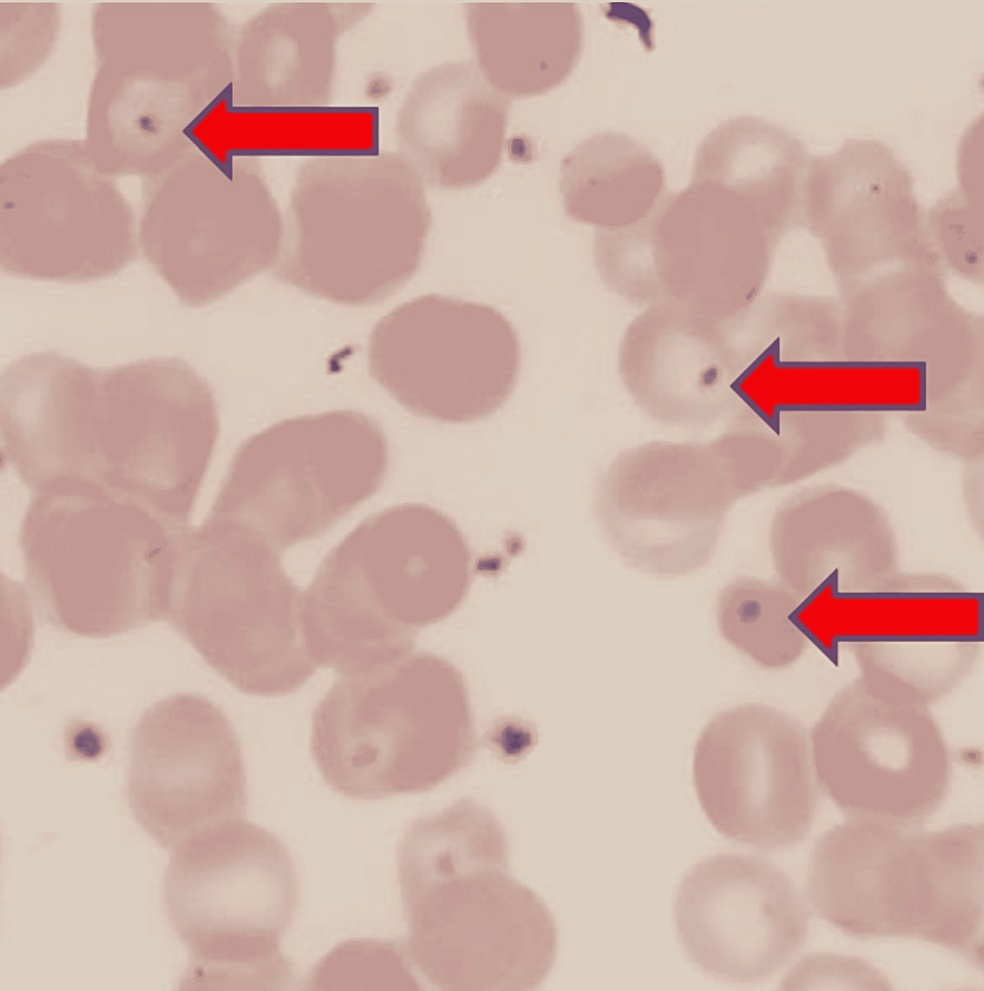
Microscopic peripheral blood smear demonstrating Howell-Jolly body.

**Figure 5:**
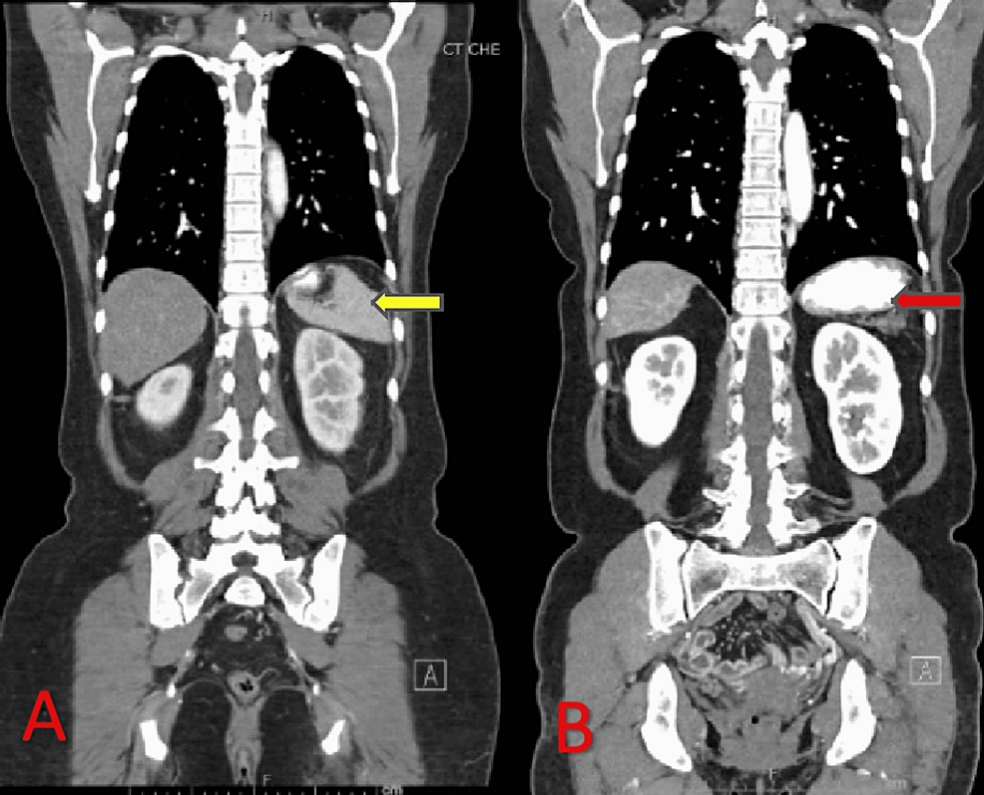
CT contrast coronal images of (A) pre-therapy and (B) post-therapy showing significantly decreased size of the spleen on the post-therapy image (red arrow) compared to the pre-therapy image (yellow arrow). CT: computed tomography

Maintenance therapy nivolumab was later discontinued after seven months (14 cycles) due to persistent elevation in the liver enzymes to four-fold the normal limit. A complete blood count (CBC) in 2017 demonstrated Howell-Jolly bodies, which progressively increased in interval CBC differential from 2017 to 2021 (Figure 4). A repeat CT scan with and without contrast of the chest, abdomen, and pelvis revealed a significant reduction in the size of the spleen and worsening metastatic disease with interval development of innumerable hypoattenuating lesions in the liver (Figure 5). Magnetic resonance imaging (MRI) abdomen revealed numerous nodules, representing the atypical appearance of metastatic disease. Repeated serial imaging demonstrated further regression of the spleen. Interventional radiology-guided liver lesion biopsy showed increased Kupffer cells and mild anisonucleosis but was negative for malignancy. Since the liver biopsy was negative for malignancy patient is currently under observation. Other causes of auto-splenectomy such as splenic infarction and sickle cell disease were ruled out. The patient received pneumococcal, meningococcal, and Haemophilus influenza vaccines. 

## Discussion

As per the American Cancer Society, melanoma accounts for 1% of all skin malignancies but is one of the leading causes of death among skin cancer [7]. The prevalence of melanoma is approximately 1.5 times more common in males than females [8]. Melanoma is a predominant disease among whites, around 20 times more common than African Americans [7]. Overall, the lifetime risk of getting melanoma is about 2.6% (1 in 38) for whites, 0.1% and 0.6% (1 in 167) for Hispanics, and 0.1% (1 in 1000) for Blacks [7]. The 10-year survival rate for patients with metastatic melanoma is less than 10% [8]. Patients with distant metastases have a five-year survival rate of 23%, making metastasis the leading cause of melanoma-associated deaths [9].

Melanoma can be divided into superficial spreading melanoma, lentigo malignant melanoma, and nodular melanoma. Superficial spreading melanoma accounts for 70%, lentigo malignant melanoma 4-15% and nodular melanoma 5% of all the melanomas [10].

It is crucial to have early detection of melanoma as it is the critical factor in lowering mortality. Diagnosis of melanoma has the advantage of the cutaneous location, which leads to early detection through non-invasive approaches. However, the gold standard for the diagnosis is pathological review via biopsy [10].

For the treatment of metastatic melanoma, dacarbazine was primarily used in the past, which had a response rate of 10-20% [11]. Immunotherapy is the new generation treatment for metastatic melanoma. Immunotherapy includes biological agents (interferons, cytokines, and granulocyte-monocyte colony-stimulating factors), vaccination strategy based on the peptide, adoptive cell therapy (lymphokine-activated killer cells, tumor-infiltrating lymphocytes), and immune checkpoint inhibitors (CTLA-4 and PD-1) [12-15]. Immune checkpoint inhibitors have drastically increased the overall survival of metastatic melanoma [16].

Since immunotherapy has increased in treating metastatic melanoma, many immune-related adverse events (irAEs) are reported. These irAEs are thought to be due to upregulation of T-cells leading to disruption of immune tolerance, ultimately causing auto-immune syndromes in various host tissues. Commonly seen irAEs with immunotherapy are thyroiditis, hypophysitis, pneumonitis, hepatitis, colitis, and a local or generalized body rash. Here we present a rare case of auto-splenectomy with the use of ipilimumab and nivolumab. Only one similar case has been reported in the published literature; a 77-year-old caucasian male was treated for metastatic melanoma with ipilimumab and nivolumab and later was found to have complete regression of the spleen [6]. In our case, a 55-year-old Caucasian female with metastatic disease was treated with four cycles of ipilimumab and nivolumab and later was switched to nivolumab maintenance therapy for 14 cycles.

Auto-splenectomy is widely seen and well documented in a patient with sickle cell disease, where sickling of the red blood cells leads to disruption of the blood supply in splenic parenchyma. Splenectomy or hyposplenism is also seen in patients with splenic infarct leading to ischemia. There is also a case reported of splenectomy in a patient with systemic lupus erythematosus (SLE) likely due to auto-immunopathy [17]. The exact mechanism of auto-splenectomy is unknown with the use of immunotherapy but could be due to auto-immunity and T-cell-related splenic cell death.

## Conclusions

We may encounter patients with immunotherapy-induced asplenia and encapsulated bacterial infections. It is crucial to create awareness among physicians of these rare irAEs. It is essential to rule out common diseases causing auto-splenectomy like sickle cell disease and splenic thrombosis. These patients should also be vaccinated against the encapsulated organisms.
